# Circular RNA ciRs-126 promotes hypoxia/reoxygenation cardiac injury possibly through miR-21

**DOI:** 10.1186/s12959-021-00355-x

**Published:** 2022-01-04

**Authors:** Changming Tan, Jianming Li, Zhaoshun Yuan, Yongxin Mu

**Affiliations:** 1 Department of Cardiovascular Surgery, The Second Xiangya Hospital, Central South University, No.139 Middle Renmin Road, 410011 Changsha, Hunan China; 2 Department of Medicine, University of California, La Jolla, CA San Diego, USA

**Keywords:** Hypoxia/reoxygenation cardiac injury, ciRs-126, miR-21, Apoptosis

## Abstract

**Background:**

This study aimed to analyze the role of circular RNA ciRs-126 in hypoxia/reoxygenation cardiac injury (H/R).

**Methods:**

Expression of ciRs-126 and miR-21 in plasma samples from patients with H/R and healthy controls was determined by RT-qPCR. Correlations were analyzed by linear regression. Overexpression of ciRs-126 and miR-21 was achieved in cardiomyocytes to explore their crosstalk. The roles of ciRs-126 and miR-21 in H/R-induced apoptosis of cardiomyocytes were analyzed using cell apoptosis assay.

**Results:**

CiRs-126 was upregulated and miR-21 was downregulated in H/R patients. They were inversely correlated across plasma samples from H/R patients. In H/R cardiomyocytes, ciRs-126 was upregulated and miR-21 was downregulated. In cardiomyocytes, ciRs-126 overexpression decreased miR-21 level and reduced the inhibitory effects of miR-21 overexpression on H/R-induced cell apoptosis.

**Conclusions:**

Circular RNA ciRs-126 may suppress miR-21 expression to promote H/R cardiac injury.

**Supplementary Information:**

The online version contains supplementary material available at 10.1186/s12959-021-00355-x.

## Background

Hypoxia/reoxygenation-induced cardiac injury (H/R), also known as myocardial ischemia or cardiac ischemia, is caused by reduced blood flow [1]. H/R is usually caused by atherosclerosis, blood clot, and coronary artery spasm [2]. With the development of H/R, heart attack, arrhythmia, and heart failure may occur, leading to a high mortality rate [3, 4]. In severe cases, 15% of patients with H/R will die before hospitalization, and 15% will die during hospitalization. Even worse, about 10% of survivors will die within one year after discharge [5, 6]. H/R is usually treated with angioplasty or bypass surgery [7], while injuries are usually irreversible. Therefore, novel treatment approaches are still needed.

Previous studies of H/R have identified considerable molecular factors involved in the initiation and development of H/R [8–10]. Increased understanding of the molecular mechanism of H/R facilitates the development of novel treatment approaches [9, 10]. Circular RNA (circRNA) is a class of circular RNAs with 3’ and 5’ ends covalently closed [11]. Previous studies have identified critical functions of circRNA in diverse pathological and physiological processes, including H/R [12]. A recent study has shown that circRNA ciRs-126 promotes acute kidney injury [13], while its role in H/R is unknown. We performed a preliminary deep sequencing analysis and observed the altered expression of ciRs-126 in H/R and its inverse correlation with miR-21, which plays protective roles in H/R [14]. This study was therefore carried out to analyze the crosstalk between ciRs-126 and miR-21 in H/R.

## Methods

### Patients and controls

This study included a total of 60 patients with H/R (H/R group, 30 males and 30 females) and 60 healthy controls (30 males and 30 females) at The Second Xiangya Hospital, Central South University from May 2019 to May 2020. All participants were at the age of 38 to 66 years, with an average of 52.1±6.7 years for patients and 52.0±6.8 years for controls. All patients were diagnosed based on the ACC/AHA/ESC/WHF practice guidelines. Table 1 lists patients’ detailed clinical information. H/R in the 60 patients was caused by the formation of blood clots in arteries. The patients were divided into 5 stage groups according to the time after infarction, namely, <4 h, 4-6 h, 6-8 h, 8-10 h, and 10-12 h. The number of H/R patients in each group was 11, 12, 11, 16, and 10, respectively. The patients with chest pain of more than 12 h and those with cardiac injury caused by factors other than H/R were excluded. All healthy controls received systemic physiological exams, and all physiological functions were within normal range. Patients with other severe diseases, such as cancers and diabetes, were excluded. This study was approved by the Ethics Committee of the Second Xiangya Hospital, Central South University. All patients and controls signed informed consent.

**Table 1:** Clinical parameters of the study subjects

Clinical parameters	H/R (*n* = 60)	Healthy subjects (*n* = 60)	*P*
Sex (male/female)	30/30	30/30	1.000
Age (years)	52.1±6.7	52.0±6.8	0.643
Tobacco	72% (43)	58% (35)	0.091
Hypertension	76% (46)	0	≠
Hypercholesterolemia	77% (46)	0	≠
Diabetes mellitus	31% (19)	0	≠
Body mass index (kg/m2)	23 ± 2,2	22 ± 1.8	0.000
Previous MI	6% (4)	0	≠
Previous CABG	1.6% (1)	0	≠
Previous PTCA	0	0	
Glucose (mmol/L)	3.1 ± 1.1	3.5 ± 0.2	0.000
High-sensitivity C-reactive protein (mg/L)	15.13 ± 6.66	1.89 ± 1.36	0.000
LDH (U/L)	481.60 ± 55.73	157.71 ± 22.94	0.000
CK-MB (U/L)	118.29 ± 55.63	10.11 ± 2.16	0.000
cTnT (*µ*g/L)	21.44 ±5.95	0	≠
Aspirin use	56% (34)	0	≠
Clopidogrel use	42% (25)	0	≠
Beta blocker use	84% (50)	0	≠
Nitrates use	77% (46)	0	≠
Angiotensin-converting-enzyme inhibitor drugs use	30% (18)	0	≠
Angiotensin receptor blocker use	11% (7)	0	≠
Calcium channel blocker use	9% (5)	0	≠
Lipid-lowering drugs	68%	0	≠

CABG: coronary artery bypass graft; PTCA: percutaneous transluminal coronary angioplasty; LDH: lactate dehydrogenase; CK-MB: creatine kinase-MB; cTnT: cardiac troponin T. P (controls VS. HR)

### Plasma and cells

To exclude the effect of dietary on ciRs-126 and miR-21 levels, blood (3 ml) was extracted into EDTA tubes from all patients and controls after they were fasted for more than 8 h and prepared as plasma samples by centrifugation for 15 min at 1200 g. The plasma samples were kept in liquid nitrogen prior to use.

Primary cardiomyocytes (C-12,810, Sigma-Aldrich, USA) were cultured in Myocyte Growth Medium (Sigma-Aldrich) in an incubator with 5% CO_2_ at 37 ºC following the instructions provided by the vendor. Cells at passages 3 to 5 were used for all experiments.

### Cell transfections

A vector expressing ciRs-126 was constructed using pcDNA3.1(+) CircRNA Mini Vector (Addgene) as the backbone. MiR-21 mimic and negative control (NC) miRNAs were purchased from Sigma-Aldrich (USA). Cardiomyocytes were transfected with 40 nM miRNA or 1 µg expression vector using Lipofectamine 2000 (Invitrogen). To perform NC experiments, cells were transfected with empty vector or NC miRNA. Control (C) cells were untransfected cells. Cells were incubated with transfection mixtures for 6 h and in fresh medium for 48 h prior to the subsequent assays.

### H/R model

After transfections (including C and NC groups), cardiomyocytes were cultured under hypoxic conditions (1% O_2_, 95% N_2_ and 5% CO_2_) for 2 h at 37 °C and in normoxic culture medium for 3 h at 37 °C to achieve reoxygenation.

### RNA preparations

RNAs from plasma and cardiomyocytes were extracted using RNAzol (Sigma-Aldrich). After being treated with DNase I (Invitrogen) for 2 h at 37 °C to remove genomic DNAs, RNA purity and integrity were examined by OD260/280 ratios and 6% urea-PAGE.

### RT-qPCRs

To synthesize cDNA samples, RNA samples with satisfactory quality were subjected to reverse transcriptions (RTs) using SS-III-RT system (Invitrogen). CiRs-126 expression was determined by qPCRs using SYBR Green Master Mix (Bio-Rad) with GAPDH, 18 S rRNA, or β-actin as the internal controls. Mature miR-21 level was determined using All-in-One™ miRNA qRT-PCR reagent kit (GeneCopoeia). To perform the reverse transcription effectively, addition of poly (A) to mature miRNAs was performed (polyA tailing method), followed by miRNA RTs and qPCRs using RPL30 and 18 S rRNA as the internal controls. All steps were completed following the manufacturer’s instructions. It is worth noting similar results were obtained using different internal endogenous controls. QPCRs were performed at 95 ºC for 1 min followed by 40 cycles of 10 s at 95 ºC and 48 s at 58 ºC.

All qPCRs were performed with three technical replicates. Ct values of target genes were used normalized to the internal controls using the 2^−ΔΔCt^ method. Supplemental Table 1 lists all primer sequences.

### Dual-luciferase assays

Dual-GloR Luciferase Assay System (Promega, USA) was used to conduct the dual-luciferase assays. In brief, miR-21 promoter was amplified using primer pair Fr-TGTAAAACGACGGCCAGT and Re-CAGGAAACAGCTATGACC and cloned into the pmirGLO vector. Cardiomyocytes were transfected with the reporter plasmid and with/without ciRs-126. After 2 days, cells were washed, digested with trypsin, and collected by centrifugation for 3 min at 5000 g. The cellular proteins were then extracted using 100 µL of lysis buffer. 50 µL of protein supernatants were added to a 96-well measuring plate and mixed in turn with 50 µL of Luciferase Reagent and Stop & Glo Reagent. The firefly luciferase activities were measured on Centro LB960 with renilla luciferase as the internal reference to correct the transfection efficiency.

### Western blot analysis

Total proteins were extracted from transfected cells. After determining protein concentration using BCA Protein Assay Kit, the same amount of proteins were separated by SDS-PAGE and transferred onto membranes. Protein levels were detected using antibodies against caspase-3 (1:1000; sc-65,497), Bax (1:1000; sc-7480), Bcl-2 (1:1000; sc-7382) and GAPDH (TA-09) (Yatai hengxin. Beijing, China), and horseradish peroxidase (HRP)-conjugated goat anti-rabbit IgG (1:1000; sc-2004, Santa Cruz, CA).

### Cell apoptosis assay

Following cell transfections and H/R modeling, cardiomyocytes were collected, washed with ice-cold PBS, and resuspended in Annexin binding buffer to reach a final density of 10^5^ cells per ml (Dojindo). After that, cells were stained with Annexin V-FITC and PI (Dojindo) in the dark for 12 min. Apoptotic cells were then analyzed by flow cytometry. In each experiment, three biological replicates were included.

### Statistical analysis

Gene expression levels in plasma samples were expressed as average values of three technical replicates and compared using unpaired t test. Data of three biological replicates of cell transfection groups were expressed as mean±SD values and compared by ANOVA Tukey’s test. *P*<0.05 was considered statistically significant.

## Results

### Altered ciRs-126 and miR-21 expressions were observed in H/R

**Fig. 1:**
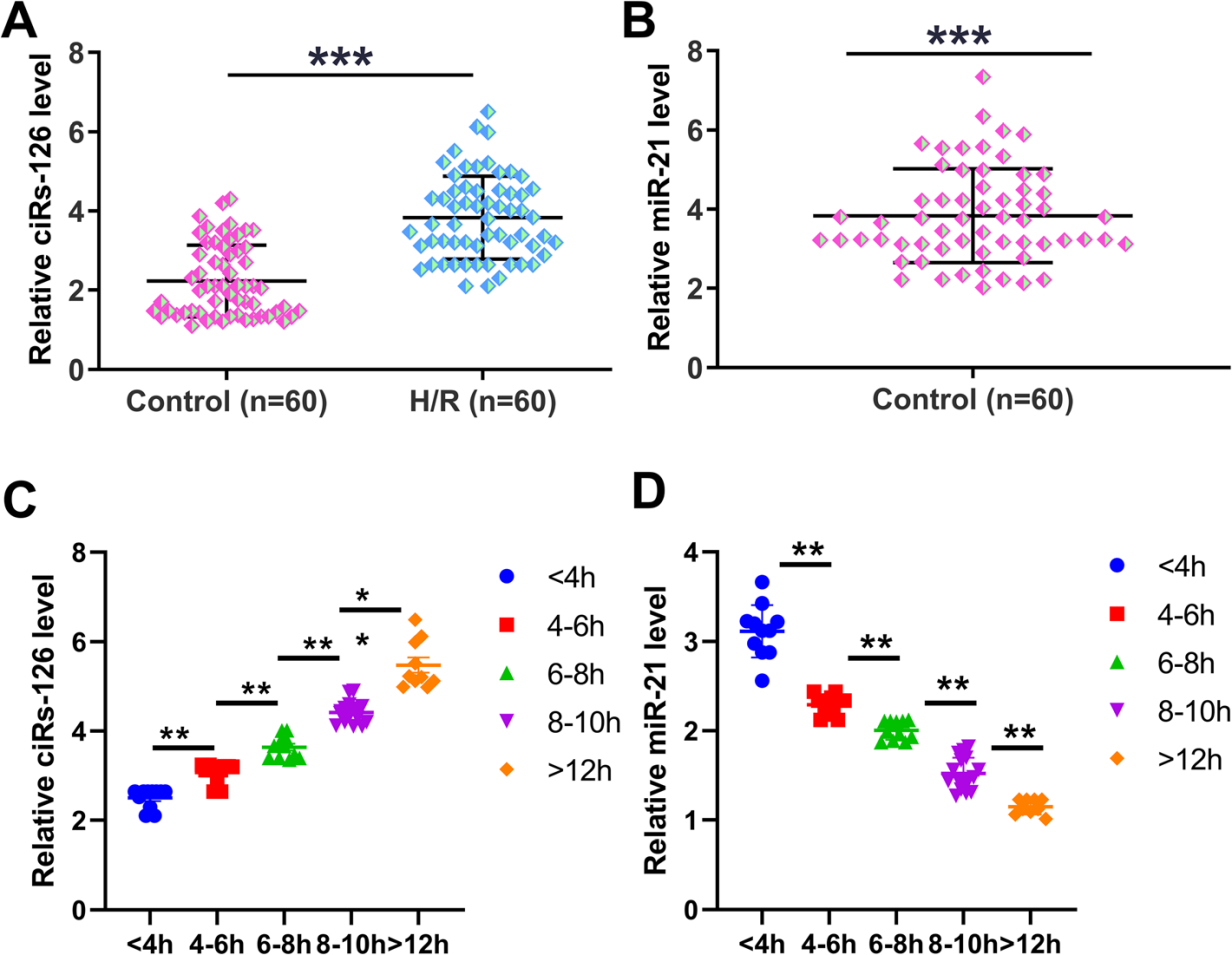
Altered expression of ciRs-126 and miR-21 was observed in H/R. To determine the expression of ciRs-126 and miR-21 in H/R, plasma samples were collected from H/R patients (*n*=60) and controls (*n*=60), followed by the determination of ciRs-126 (**A**) and miR-21 (**B**) expression by RT-qPCR. The expression of ciRs-126 and miR-21 in patients at different H/R stages were detailed in (**C**) and (**D**). Gene expression levels in plasma samples were expressed as average values of triplicate technical replicates. “1” indicated the related expression compared to GAPDH. **, *p*<0.05; ***, *p*<0.001

To determine the expression of ciRs-126 and miR-21 in H/R, plasma samples were collected from H/R patients (*n*=60) and controls (*n*=60), and ciRs-126 and miR-21 expression were determination by RT-qPCR. Compared to the control group, ciRs-126 was significantly overexpressed in H/R group (Fig. 1 A, *p*<0.001), while miR-21 was significantly under-expressed in H/R group (Fig. 1B, *p*<0.001). The expression of ciRs-126 and miR-21 were positively and negatively correlated with different stages of H/R patients, respectively (Fig. 1 C and Fig. 1D, *p*<0.05). Furthermore, the correlation between the expression of Cir-126, CK-MB and cTnT in H/R samples were also performed. The results indicate that the expression of Cir-126 was correlated with the amount of CK-MB and cTnT(supplemental figure-1). Our data suggested that altered expression of ciRs-126 and miR-21 may be involved in H/R.

### A significant and inverse correlation between ciRs-126 and miR-21 was observed across plasma samples from H/R patients

**Fig. 2:**
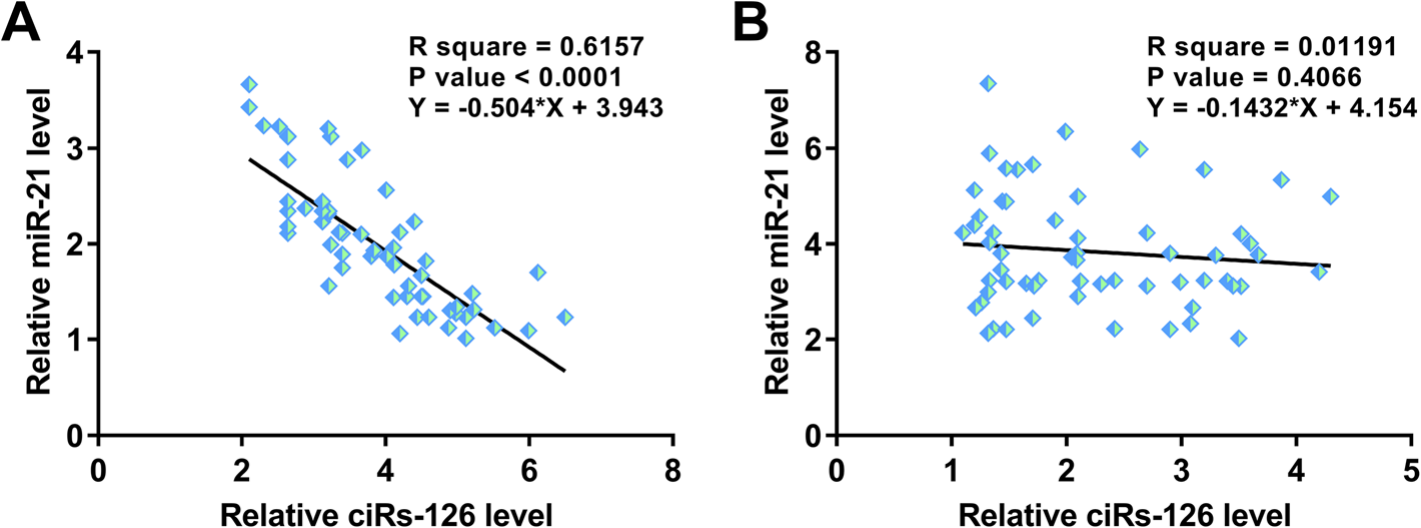
A significant and inverse correlation between ciRs-126 and miR-21 was observed across plasma samples from H/R patients. Correlations between ciRs-126 and miR-21 across plasma samples from both H/R patients (**A**) and healthy controls (**B**) were analyzed by linear regression

Correlations between ciRs-126 and miR-21 across plasma samples from both H/R patients and healthy controls were analyzed by linear regression. It was observed that ciRs-126 and miR-21 were inversely and significantly correlated with each other across plasma samples from H/R patients (Fig. 2 A). However, no significant correlation between ciRs-126 and miR-21 was observed across plasma samples from the healthy controls (Fig. 2B). Therefore, ciRs-126 and miR-21 may interact with each other in H/R, and their crosstalk is likely mediated by certain H/R-related pathological factors.

### CiRs-126 overexpression decreased miR-21 expression in H/R cell model but not in non H/R treated cells

**Fig. 3:**
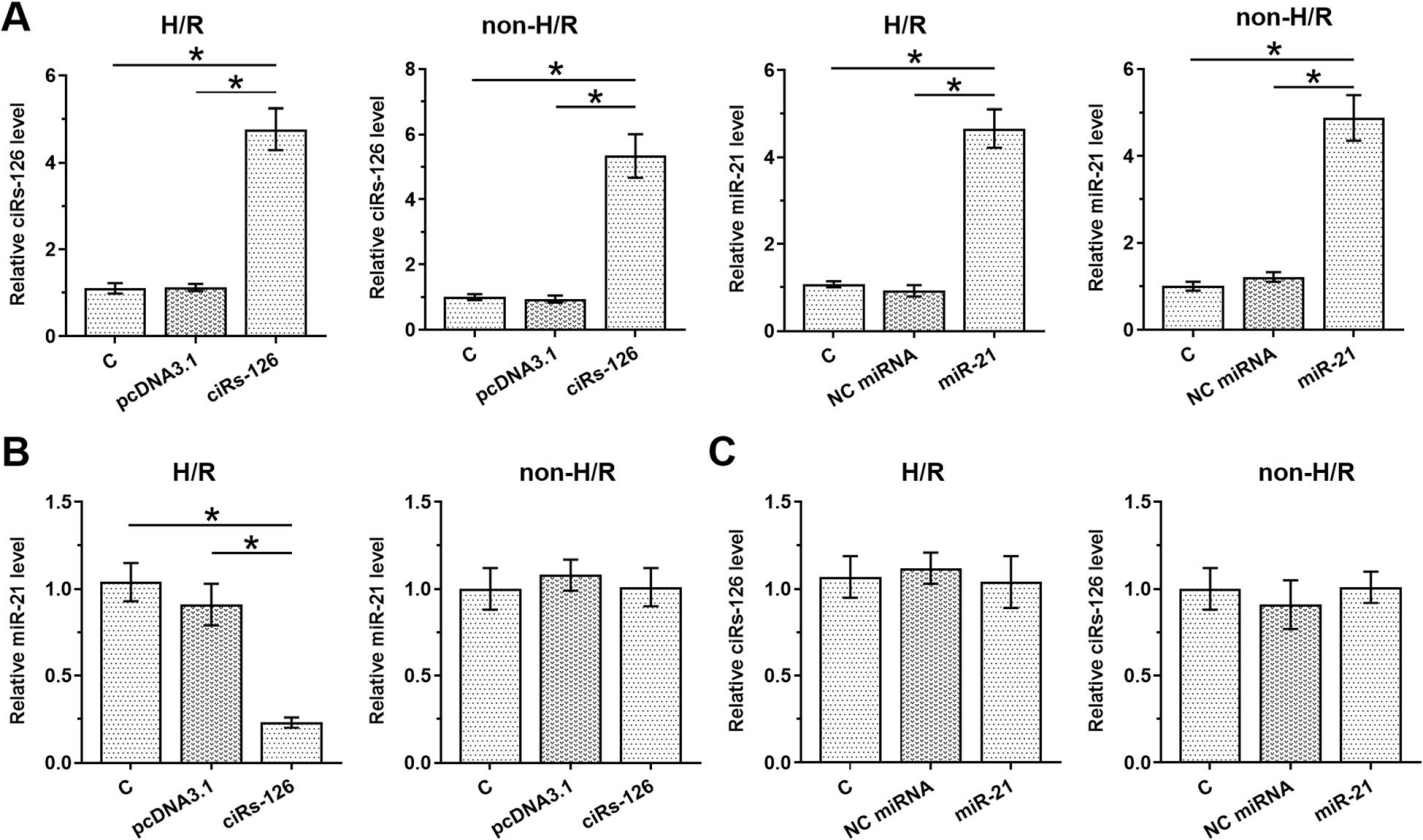
CiRs-126 overexpression decreased miR-21 expression in H/R cell model but not in non H/R treated cells. To explore the crosstalk between ciRs-126 and miR-21, ciRs-126 expression vector or miR-21 mimic was transfected into H/R-treated cardiomyocytes and non H/R treated cardiomyocytes. Overexpression of ciRs-126 and miR-21 was confirmed by RT-qPCR (**A**). The effects of ciRs-126 overexpression on miR-21 expression (**B**) and the effects of miR-21 overexpression on ciRs-126 (**C**) in both H/R-treated cardiomyocytes and non H/R treated cardiomyocytes were also analyzed by RT-qPCR. The cirRNA or miRNA expression in “C” of untreated cells was set as “1” and used to normalize expressions in other groups. Data of three biological replicates of cell transfection groups were expressed as mean±SD. *, *p*<0.05

Our bioinformatics analysis and a previous study have suggested that ciRs-126 and miR-21 could only form an 8-base pairing. Hence it may not be sufficient for ciRs-126 to sponge miR-21. Luciferase assay was then performed to investigate the promoter activity of miR-21 influenced by ciRs-126 in cardiomyocytes and the results showed no difference between ciRs-126 transfection and control groups (Supplemental figure-2). To explore the crosstalk between ciRs-126 and miR-21, ciRs-126 expression vector or miR-21 mimic was transfected into H/R-treated cardiomyocytes and non H/R treated cardiomyocytes. Overexpression of ciRs-126 and miR-21 was confirmed by RT-qPCR (Fig. 3 A, *p*<0.05). It was found that ciRs-126 overexpression decreased miR-21 level in H/R cell model, but not in non H/R treated cells (Fig. 3B, *p*<0.05). However, miR-21 overexpression failed to significantly alter ciRs-126 expression in both H/R-treated and untreated cardiomyocytes (Fig. 3 C). Therefore, ciRs-126 is likely a conditional dependent upstream inhibitor of miR-21 in H/R.

### ciRs-126 overexpression attenuated the inhibitory effect of miR-21 on H/R-induced cardiomyocyte apoptosis

**Fig. 4:**
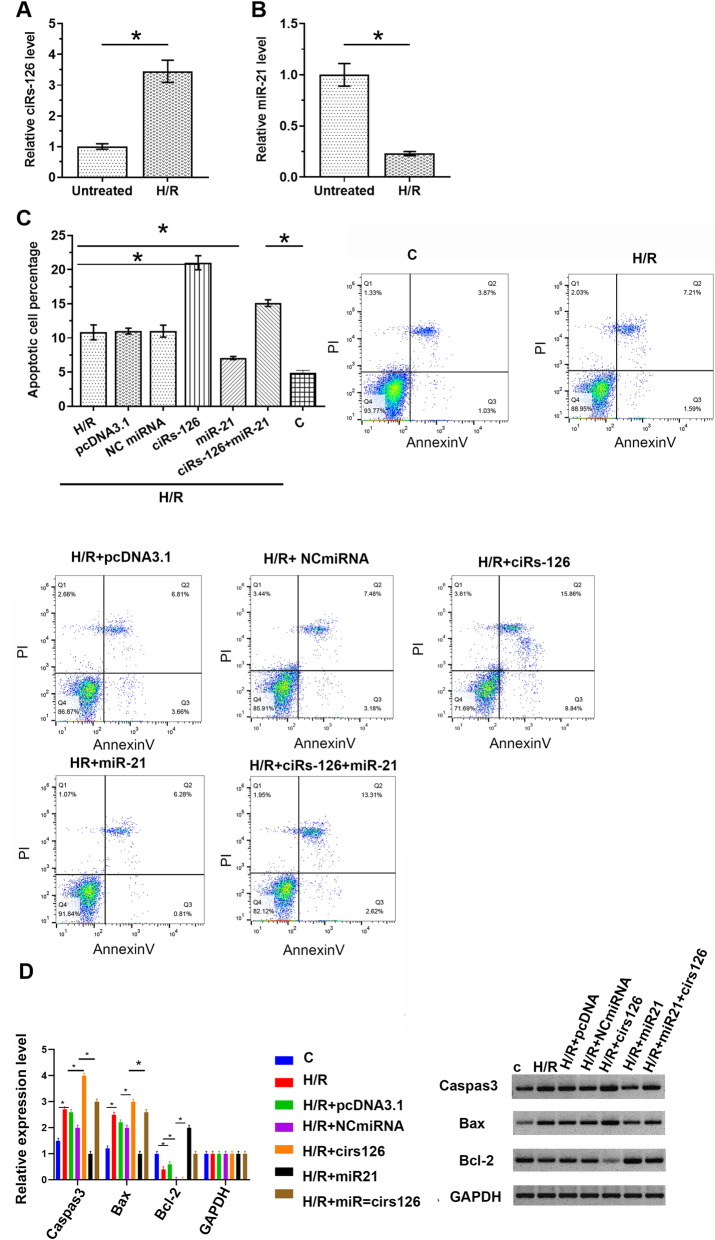
CiRs-126 overexpression attenuated the inhibitory effect of miR-21 on H/R-induced cardiomyocyte apoptosis. Cardiomyocytes were subjected to H/R treatment, followed by the measurement of expression levels of ciRs-126 (**A**) and miR-21 (**B**) by RT-qPCR. Cell apoptosis assay was performed to analyze the roles of ciRs-126 and miR-21 in regulating cardiomyocyte apoptosis induced by H/R (**C**). Western blot analysis for caspase 3, Bax, and Bcl-2 from cardiomyocytes induced by H/R (**D**). Data of three biological replicates of cell transfection groups were expressed as mean±SD. *, *p*<0.05

CiRs-126 and miR-21 levels in H/R-treated cardiomyocytes were measured by RT-qPCR. The results showed that H/R cell model exhibited significantly overexpressed ciRs-126 (Fig. 4 A, *p*<0.05) and under-expressed miR-21 compared to untreated cells (Fig. 4B, *p*<0.05). Thus, the roles of ciRs-126 and miR-21 in regulating H/R-induced cardiomyocyte apoptosis was analyzed using cell apoptosis assay. CiRs-126 overexpression significantly increased cell apoptosis, while miR-21 overexpressed decreased cells apoptosis. In addition, ciRs-126 overexpression reduced the inhibitory effects of miR-21 overexpression on H/R-induced cell apoptosis and apoptosis-related protein expression (Fig. 4 C-D, *p*<0.05).

## Discussion

This study mainly investigated the involvement of ciRs-126 and miR-21 in H/R and explored their crosstalk. We found that ciRs-126 was overexpressed in H/R. In addition, ciRs-126 could downregulate miR-21 to promote the apoptosis of cardiomyocytes induced by H/R.

The function of ciRs-126 has only been studied in acute kidney injury and polycystic ovarian syndrome[13, 15]. It was observed that ciRs-126 was significantly overexpressed in patients with acute kidney injury and could sponge miR-126-5p to participate in disease progression[13]. In polycystic ovarian syndrome, ciRs-126 is downregulated and targets miR-21-PDCD4-ROS axis to suppress ovarian granulosa cell proliferation[15]. This study, for the first time, reported the upregulation of ciRs-126 in H/R. In addition, ciRs-126 overexpression significantly increased the apoptosis of cardiomyocytes induced by H/R. Therefore, ciRs-126 may promote H/R injuries by promoting cell apoptosis. Moreover, H/R treatment increased ciRs-126 expression level in cardiomyocytes. Therefore, H/R inducible ciRs-126 promotes cardiac injuries by increasing cell apoptosis.

Huang et al. recently showed that miR-21 is significantly under-expressed in H/R model and miR-21 overexpression suppressed cell apoptosis and autophagic activity in cardiac injury, possibly through AKT /mTOR pathway[14]. Consistently, our study observed the downregulation of miR-21 in patients with H/R and cardiomyocytes after H/R treatment[14]. In addition, miR-21 overexpression decreased the apoptosis of cardiomyocytes induced by H/R. Therefore, our study further confirmed the protective role of miR-21 in H/R.

It has been reported that ciRs-126 could sponge miR-21 to suppress the role of miR-21 in the proliferation and apoptosis of ovarian granulosa cell proliferation[15]. However, based on our bioinformatics analysis and this study, ciRs-126 and miR-21 can only form an 8-base pairing, which may not be sufficient for ciRs-126 to sponge miR-21. In this study, luciferase assay results showed that there was no direct regulation between miR-21 and ciRs-126. We showed that ciRs-126 could downregulate miR-21 in H/R models but not in cells without H/R treatment. Therefore, H/R-related factors may mediate the crosstalk between ciRs-126 and miR-21. The results indicated the existence of other conditional dependent regulation mechanisms for ciRs-126 and miR-2.Our result was consistent with previous in acute kidney injury cells study, namely, Circular RNA sponge of miR-126 (or ciRs-126) was most altered compared to healthy controls and disease controls[16]. No matter in the H/R or acute kidney injury cells, the sponge between ciRs-126 and miR-21 was enhanced in the hypoxic cells. One likely explanation is that miR-126 has been shown to control hypoxia signaling and ciRs-126 may be part of signaling cascades involving LRIG( the liner form of ciRs-126) and miR-126. The associated signaling pathways include EGFR family, tyrosine-protein kinase Met, and RET proto-oncogene[17, 18]. There may be many signaling cross-talks and feedback loops to regulate the homeostasis between ciRs-126 and miR-126. More detailed works need perform in future. Furthermore,the levels of ciRs-126 were also closly co-related to those of CK-MB and cTnT, which indicate the dead/dying cardiomyocytes, showed that the ciRs-126 was also released from such cells and indicated the cell injury caused by hypoxia. Taken together, the conditional existence of ciRs-126/miR-21 in H/R cardiomyocytes indicates that ciRs-126 up-regulation and miR-21 down-regulation might be a pair of potential biomarkers for myocardial ischemia, especially at the early stage. More studies are needed in the future.

## Conclusions

In conclusion, ciRs-126 is overexpressed in H/R and may downregulate miR-21 to promote the apoptosis of cardiomyocytes induced by H/R.

## Supplementary information

**Additional file 1.**

Additional file 2: **Supplement Fig. 1.** The correlation analysis between the expression of Cir-126, CK-MB and cTnT in H/R samples. Linear regression analysis results of the expression of Cir-126 and the concentration of CK-MB (**A**)and cTnT(**B**) in H/R samples. The Pearson correlation coefficient is 0.910 and 0.905, respectively.

Additional file 3: **Supplement Fig. 2.** Luciferase Assay. Luciferase activity detection by dual-luciferase reporter assay following cardiomyocytes treated with/without H/R. Cir-126 and reporter vector of miR-21 promoter were then transfected, the luciferase activity (1.01±0.02 VS 0.93±0.02;10.84±0.07 VS 0.16±0.01, *p*>0.5, df=2) was then examined .Students’t test was performed to compare different groups. All data were presented as mean±SD in each figure. *n* =3.

## Data Availability

The datasets used and/or analyzed during the current study are available from the corresponding author on reasonable request.

## Abbreviations

H/R: Hypoxia/reoxygenation cardiac injury
circRNA: Circular RNA
RTs: Reverse transcriptions
