# Supplementary material for: Circular RNA ciRs-126 promotes hypoxia/reoxygenation cardiac injury possibly through miR-21
Source: Thromb J. 2022 Jan 4;20:2. doi: 10.1186/s12959-021-00355-x (PMC8725357; doi:10.1186/s12959-021-00355-x)
Supplement: Supplementary file 1 — Additional file 1. [file 12959_2021_355_MOESM1_ESM.doc]

| **Supplemental Table S1.** Primer pairs | |
| --- | --- |
| **RNA transcript** | **Primer Pairs** |
| *circular RNA sponge for* ciRs-126 (*hsa_circ_0003266)* | Fwd: AGTTGACAGCGGTACCATCC  Rev: GTAGGTTCGGCAAGTCCTCA |
| *miR-21* | Fwd: ACACTCCAGCTGGGTAGCTTATCAGACTGA  Rev: TGGTGTCGTGGAGTCG |
| *U6* | Fwd: CGCTTCGGCAGCACATATACTAAAATTGGAAC  Rev: GCTTCACGAATTTGCGTGTCATCCTTGC |
| 18s rRNA | Fwd: GTAACCCGTTGAACCCCATT  Rev: CCATCCAATCGGTAGTAGCG |
