# Supplementary figures and images for: Circular RNA ciRs-126 promotes hypoxia/reoxygenation cardiac injury possibly through miR-21
Source: Thromb J. 2022 Jan 4;20:2. doi: 10.1186/s12959-021-00355-x (PMC8725357; doi:10.1186/s12959-021-00355-x)

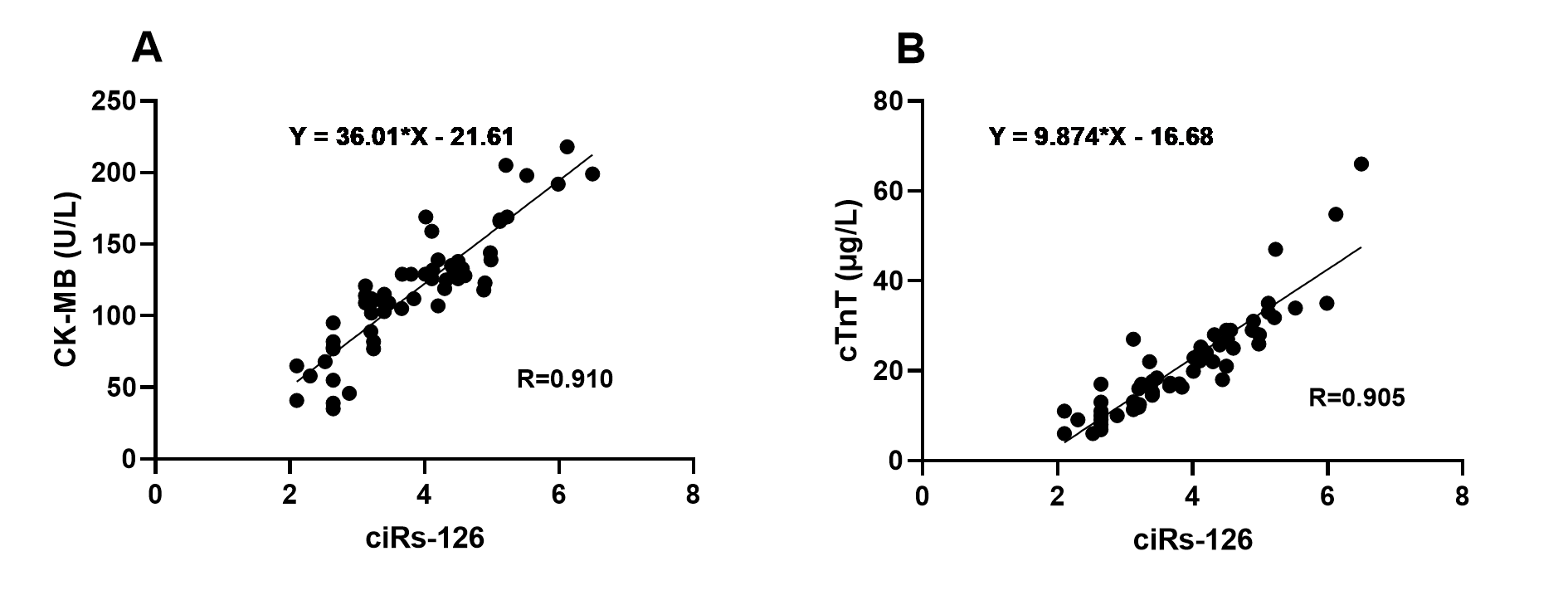

Supplement: Supplementary file 2 — Supplement Fig. 1. The correlation analysis between the expression of Cir-126, CK-MB and cTnT in H/R samples. Linear regression analysis results of the expression of Cir-126 and the concentration of CK-MB (A)and cTnT(B) in H/R samples. The Pearson correlation coefficient is 0.910 and 0.905, respectively. [file 12959_2021_355_MOESM2_ESM.tif]

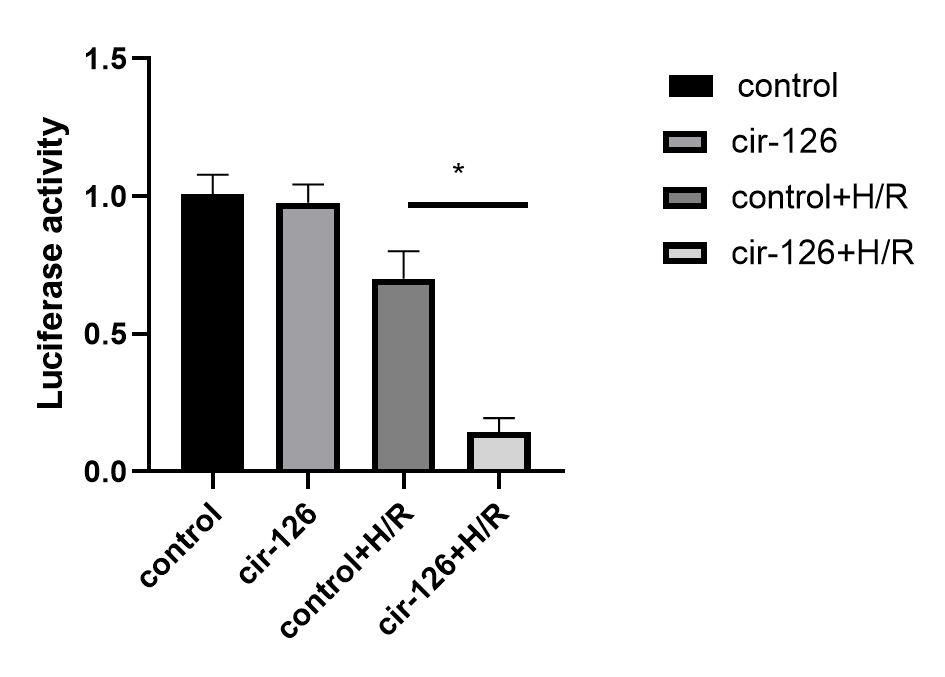

Supplement: Supplementary file 3 — Supplement Fig. 2. Luciferase Assay. Luciferase activity detection by dual-luciferase reporter assay following cardiomyocytes treated with/without H/R. Cir-126 and reporter vector of miR-21 promoter were then transfected, the luciferase activity (1.01±0.02 VS 0.93±0.02;10.84±0.07 VS 0.16±0.01, p>0.5, df=2) was then examined .Students’t test was performed to compare different groups. All data were presented as mean±SD in each figure. n =3. [file 12959_2021_355_MOESM3_ESM.tif]
